# Supplementary material for: Patterns of Intron Gain and Loss in Fungi
Source: PLoS Biol. 2004 Nov 30;2(12):e422. doi: 10.1371/journal.pbio.0020422 (PMC532390; doi:10.1371/journal.pbio.0020422)
Supplement: Table S1 — Also available at http://genes.mit.edu/NielsenEtAl/. (4.3 MB ZIP). [file pbio.0020422.st001.zip › NielsenEtAl/html/1202.html]

AN1287.1.NCU05633.1.MG07912.1.FG09294.1


```
 CLUSTAL W (1.82) Multiple Sequence Alignments - Introns Inserted


Sequence 1: NCU05633.1	431 aa
Sequence 2: MG07912.1	423 aa
Sequence 3: FG09294.1	400 aa
Sequence 4: AN1287.1	427 aa
Alignment Length: 439 aa
Number Identitical Residues: 234 aa
Alignment Score (without introns) 9448


MG07912.1 	-MAT~ALVNR~SNPAALPAVRRTIAAAAGAIGTTSTPTTSLVLSSPRQQQRNRQQQRRTF
NCU05633.1	MTMT~AATTQ~RALAPLARHQQRLLTSATATSARSLSTRRRALLPASTTAPTSSSTAVSN
FG09294.1 	---M~AMSLA~RVALRAPQSQSRFAAAAATITARTIVAYGRHFSTTNQALIPS-------
AN1287.1  	---M0CSTLL2SLLPAMELASKQTARLLRPSTVRLHPGLPSAISTTQTALRRRNISTTPW
          	     .                       .  .         : ..       .    . 

MG07912.1 	LEQAGSDASKAGSAGAGPGGRGAIPTYFQ-RERLPANTVIRFVPQQTAWVVERMGKFHRI
NCU05633.1	FHTSANHAIPTG----GFGGGGGIPTYFQ-KPSLPANTIIRFVPQQTAWIVERMGKFNRI
FG09294.1 	----------------GFGSP-IVPSYFS-KPRLPANTVIRFVPQQTAWIVERMGKFNRI
AN1287.1  	RNAPESSLLSLA----SSSSSGSPPTYFSNRQTLPMNTVVRFVPQQTAWIVERMGKFHRI
          	   . .     .    . .. .  *:**..:  ** **::*********:*******:**

MG07912.1 	LEPGLAILVPFLDRIAYVKSLKEVAIEIPSQSAITADNVTLELDGVLYTRVFDAYKAS2Y
NCU05633.1	LQPGLAILIPFIDRIAYVKSLKEVAHEIPSQSAITADNVTLELDGVLYTRVFDAYKAS2Y
FG09294.1 	LEPGLAVLVPFIDRIAYVKSLKEVAIEIPSQSAITADNVTLELDGVLFTRVFDAYKAS2Y
AN1287.1  	LEPGLAILVPFLDRIAYVKSLKESAIEIPSQNAITADNVTLELDGVLYTRVFDAYKAS2Y
          	*:****:*:**:*********** * *****.***************:********** *

MG07912.1 	GVEDAEYAISQLAQTTMRSEIGQLTLDHVLKERAALNTNITAAINEAAQAWGVTCLRYEI
NCU05633.1	GVEDAEYAISQLAQTTMRSEIGQLTLDHVLKERAALNTNITAAINEAAQAWGVTCLRYEI
FG09294.1 	GVEDAEYAISQLAQTTMRSEIGQLTLDHVLKERAALNTNITAAINDAAEAWGVTCLRYEI
AN1287.1  	GVEDAEYAISQLAQTTMRSEIGQLTLDHVLKERAMLNTNITQAINEAAQAWGVTCLRYEI
          	********************************** ****** ***:**:***********

MG07912.1 	RDIHAPTAVVEAMHRQVTAERSKRAEILDSEGQRQSAINIAEGRKQSVILASEALKAEKI
NCU05633.1	RDIHAPKPVVEAMHRQVTAERSKRAEILESEGQRQSAINIAEGKKQSVILASEAMKAEQI
FG09294.1 	RDIHAPGAVVEAMHRQVTAERSKRAEILESEGQRQSAINIAEGKKQSVILASEALRAERI
AN1287.1  	RDIHAPDGVVEAMHRQVTAERSKRAEILDSEGQRQSAINIAEGRKQSVILASEADRIERI
          	******  ********************:**************:********** : *:*

MG07912.1 	NRAEGEAEAILLKARATAQGIDQVARSMAEGKQAAQGAVNLSVAEKYVEAFGKLAKEGTA
NCU05633.1	NRASGQAEAIRLKAVATAGGIEAVARAIAEGQGAAQNAVSLSVAEKYVDAFGKLAKEGTA
FG09294.1 	NEADGEAEAIRLKASATAQGIDAVSESIIRGDAGAQAAVSLRVAEKYVDAFGKLARESTA
AN1287.1  	NRANGEAAAIRAKAEATAKAIETVAQAIEAGQANAHGAISLNIAEKYVEAFGKLAHEGTA
          	*.*.*:* **  ** *** .*: *:.::  *.  *: *:.* :*****:******:*.**

MG07912.1 	VVVPGNVGDIGGMIATALSVYGKVGAVQGQANG-------GAVAKVEEATVSAPGTGDAV
NCU05633.1	VVVPGNVGDIGGMIATALSVYGKVGDAQAKAMAKQLLEKQQGEQQEADSQKALPGGGVAK
FG09294.1 	VVVPGNVGDISGMIATGLSVFGKVGQAQAQTMAKSLVEPKGDSQTAELEGQAKPDVKETV
AN1287.1  	VVVPGNMGDLGGMIANAMAVYGKVSGSQARTIAAKALG-VQDPQTTEKTPEVKPNGKRDV
          	******:**:.****..::*:***.  *.:: . .                  *.     

MG07912.1 	KDS----IVADFDKATKDR---
NCU05633.1	QESPVDDIVKGFDQATKQR---
FG09294.1 	LES--------FNQATSKR---
AN1287.1  	AEG----ILEGFEQTRREEEKI
          	 :.       .*:::  ....
```
